# Science and Instinct in Diagnosis

**Published:** 1901-02-02

**Authors:** 


### SCIENCE AND INSTINCT IN DIAGNOSIS.

It is a little difficult for the modern physician, and perhaps still more difficult for the modern surgeon, to imagine how his forefathers got on without all the paraphernalia with which in these days we delight to surround ourselves in the way of instrument of precision and methods of research, and some, no doubt, would shrug their shoulders and suggest that without either stethoscopes, microscopes, or thermometers these old gentlemen of bygone days must have put in a good deal of humbug to fill up their time. It may be doubted, however, whether the excessive trust in instrumental aids and in the dicta of the laboratory professors which characterises the up-to-date physician of the present period is altogether good, and whether a little bit of that unreasoning instinct which leads a man to think a thing without knowing why he thinks it, to come to a conclusion as to the nature of a patient's ailment without any consciousness of the mental processes which have led him to it, would not often save even the scientific observers of to-day from many a blunder into which our forefathers with their less exact methods would never have stumbled. It is obvious enough that, take things all round, modernity has its advantages. No one can doubt that laboratories, instruments, and the like are good and useful: but for all that no one can avoid seeing that the very confidence we have in these aids tends to beget a dependence upon them, and a neglect of what for want of a more precise term we may call our "instinctive impressions;" and this is a loss. A recent writer, commenting upon the occasional failure of the stethoscope to discover even well marked tuberculous cavities in the lungs, points out that the stethoscope is not the only instrument of supposed precision in regard to which there is need for caution, and that the tendency to trust implicitly to the revelations of such instruments as the laryngoscope, the microscope, and the ophthalmoscope, and to hold that from their verdicts there is no appeal, is one to be resisted, especially when these verdicts depend upon negative evidence. Take, for example, the case of cancer and its early diagnosis. Does the microscope ever correctly show a growth to be cancerous which any well skilled observer, using his naked eye and availing himself of the history of the case, that is using merely all non-instrumental means, need be in doubt about? On the other hand in how many hundreds of cases has not a negative result from microscopical examination led to an operation being deferred—much to the loss of the patient—which would otherwise have been done at once? The same again may be said, and even more strongly, of the negative results of bacteriological examination. If the stethoscope reveals to us certain sounds, if the ophthalmoscope or the laryngoscope show abnormal appearances, or if our bacteriological friends declare that a given case shows the presence of certain bacilli, there is at least something positive to go upon, and these things must be set alongside of the other symptoms, and with them used to interpret the problem presented by the case. But because these instruments and methods of precision give a negative answer, because we are told the appearances are "normal," or that "no pathogenic organisms" are to be found, we have no right to speak comfortably to our patients when our "instinct" and the rough-and-ready but none the less valuable methods employed by our grandfathers tell us that there is something wrong. Positive evidence from whatever source is always valuable and must be explained. Negative evidence, however respectable its origin, it is safest to neglect.

